# Sesamin prevents decline in exercise capacity and impairment of skeletal muscle mitochondrial function in mice with high‐fat diet‐induced diabetes

**DOI:** 10.1113/EP085251

**Published:** 2015-10-01

**Authors:** Shingo Takada, Shintaro Kinugawa, Shouji Matsushima, Daisuke Takemoto, Takaaki Furihata, Wataru Mizushima, Arata Fukushima, Takashi Yokota, Yoshiko Ono, Hiroshi Shibata, Koichi Okita, Hiroyuki Tsutsui

**Affiliations:** ^1^ Department of Cardiovascular MedicineHokkaido University Graduate School of MedicineSapporoJapan; ^2^ Institute for Health Care ScienceSuntory Wellness LtdOsakaJapan; ^3^ Department of Sport EducationHokusho UniversityEbetsuJapan

## Abstract

**New Findings:**

- **What is the central question of this study?**
  Our aim was to examine whether sesamin can prevent a decline in exercise capacity in high‐fat diet‐induced diabetic mice. Our hypothesis was that maintenance of mitochondrial function and attenuation of oxidative stress in the skeletal muscle would contribute to this result.
- **What is the main finding and its importance?**
  The new findings are that sesamin prevents the diabetes‐induced decrease in exercise capacity and impairment of mitochondrial function through the inhibition of NAD(P)H oxidase‐dependent oxidative stress in the skeletal muscle. Sesamin may be useful as a novel agent for the treatment of diabetes mellitus.

**Abstract:**

We previously reported that exercise capacity and skeletal muscle mitochondrial function in diabetic mice were impaired, in association with the activation of NAD(P)H oxidase. It has been reported that sesamin inhibits NAD(P)H oxidase‐induced superoxide production. Therefore, we examined whether the antioxidant sesamin could prevent a decline in exercise capacity in mice with high‐fat diet (HFD)‐induced diabetes. C57BL/6J mice were fed a normal diet (ND) or HFD, then treated or not with sesamin (0.2%) to yield the following four groups: ND, ND+Sesamin, HFD and HFD+Sesamin (*n* = 10 each). After 8 weeks, body weight, fat weight, blood glucose, insulin, triglyceride, total cholesterol and fatty acid were significantly increased in HFD compared with ND mice. Sesamin prevented the increases in blood insulin and lipid levels in HFD‐fed mice, but did not affect the plasma glucose. Exercise capacity determined by treadmill tests was significantly reduced in HFD mice, but almost completely recovered in HFD+Sesamin mice. Citrate synthase activity was significantly decreased in the skeletal muscle of HFD mice, and these decreases were also inhibited by sesamin. Superoxide anion and NAD(P)H oxidase activity were significantly increased in HFD mice compared with the ND mice and were ameliorated by sesamin. Sesamin prevented the decline in exercise capacity in HFD‐induced diabetic mice via maintenance of mitochondrial function, fat oxidation and attenuation of oxidative stress in the skeletal muscle. Our data suggest that sesamin may be useful as a novel agent for the treatment of diabetes mellitus.

## Introduction

Patients with metabolic syndrome and type 2 diabetes show reduced exercise capacity and mitochondrial dysfunction in the skeletal muscle (Regensteiner *et al*. 2005; Mogensen *et al*. 2007; Yokota *et al*. 2011, 2013). Abnormalities in skeletal muscle energy metabolism are the key factor in reduced exercise capacity (Okita *et al*. 1998; Yokota *et al*. 2011, 2013). Moreover, this feature has been reported to be an independent predictor of mortality (Wei *et al*. 2000). We previously reported that the reduced exercise capacity and impaired skeletal muscle mitochondrial function in mice with high‐fat diet (HFD)‐induced type 2 diabetes were due to enhanced oxidative stress via the activation of NAD(P)H oxidase (Yokota *et al*. 2009; Takada *et al*. 2013, 2014; Kinugawa *et al*. 2015). Another study reported that NAD(P)H oxidase‐induced superoxide anion (O_2_·^−^) production was increased in the skeletal muscle of mice with insulin resistance‐induced diabetes (Bonnard *et al*. 2008). The NAD(P)H oxidase‐induced enhancement of oxidative stress has also been demonstrated in skeletal muscle from patients with type 2 diabetes (Roberts *et al*. 2006). In our previous studies, angiotensin II type 1 receptor blocker or an insulin‐sensitizing drug ameliorated the activation of NAD(P)H oxidase and partly improved the limited exercise capacity (Takada *et al*. 2013, 2014). NAD(P)H oxidase activity can be increased by high fatty acid levels and activation of the renin–angiotensin system, as well as by high glucose, insulin and insulin resistance levels (Yang & Kahn, 2006; Takada *et al*. 2013, 2014; Kadoguchi *et al*. 2015). Therefore, the activation of NAD(P)H oxidase by activation of the renin–angiotensin system and/or insulin resistance plays an important role in the limited exercise capacity of HFD‐induced diabetic mice.

Sesamin, one of the lignans found in sesame seeds and oil, has multiple biological functions (Nakano *et al*. 2006, 2008; Hong *et al*. 2013). It has been reported that sesamin decreases blood glucose, insulin and lipid levels in type 2 diabetic mice (Hong *et al*. 2013). Sesamin also inhibits NAD(P)H oxidase‐induced O_2_·^−^ production in the aorta in rats administered deoxycorticosterone acetate and salt (Nakano *et al*. 2006). Furthermore, a sesamin metabolite (SC‐1; (7α,7′α,8α,8′α)‐3,4‐dihydroxy‐3′,4′‐methylenedioxy‐7,9′:7′,9‐diepoxylignane) strongly inhibited xanthin/xanthine oxidase‐induced O_2_·^−^ production (Nakai *et al*. 2003; Nakano *et al*. 2006, 2008). Given that sesamin has antioxidant effects, we hypothesized that it may have a favourable effect on mitochondrial function, preventing the decline in exercise capacity in HFD‐induced diabetic mice by inhibiting NAD(P)H oxidase‐induced production of reactive oxygen species. We therefore investigated whether sesamin could prevent the activation of NAD(P)H oxidase and decline in exercise capacity in HFD‐induced diabetic mice.

## Methods

### Experimental animals

Male C57BL/6J mice were housed in an animal room in controlled conditions on a 12 h–12 h light–dark cycle. Mice were fed either a normal diet (ND) containing 4.2% fat and 54.6% carbohydrate or an HFD (HFD32) containing 32.0% fat and 29.4% carbohydrate for 8 weeks. Mice were further divided into groups with or without the addition of sesamin (0.2%) to their ND or HFD diet. Sesamin was prepared from refined sesame seed oil and purified as previously described (Fukuda *et al*. 1986). The quantities of food consumed by each mouse (2.4‒2.5 g day^−1^ per mouse) and body weights were monitored every week (data not shown). The dose of sesamin in the present study was chosen on the basis of previous studies (Ashakumary *et al*. 1999; Ide *et al*. 2001 *a*). The present study thus had the following four treatment groups: (i) ND; (ii) ND+Sesamin; (iii) HFD; and (iv) HFD+Sesamin (*n* = 10 for each group). These assignment procedures were performed using numerical codes to identity the animals. All procedures and animal care were approved by our institutional animal research committee and conformed to the Animal Care Guideline for the Care and Use of Laboratory Animals at Hokkaido University Graduate School of Medicine.

Blood samples were collected from the inferior vena cava before the mice were killed, under deep general anaesthesia induced with tribromoethanol‐amylene hydrate [Avertin; 2.5% w/v, 250 mg (kg body weight)^−1^, i.p.] (Sigma‐Aldrich, St Louis, MO, USA). Epididymal fat and unilateral hindlimb skeletal muscles (quadriceps, gastrocnemius and soleus) were then excised and weighed. We used only the gastrocnemius muscle for mitochondrial function and biochemical analyses in all experiments (*n* = 6–10 for each assay).

In the *in vitro* study, we used mouse C2C12 myotubes and measured NAD(P)H oxidase activity (*n* = 10–11 for each group).

### Blood pressure measurements

Systemic blood pressure and heart rate were measured using the tail‐cuff method (BP‐98A; Softron, Tokyo, Japan) without anaesthesia.

### Biochemical measurements

Plasma insulin, total cholesterol, triglyceride and non‐esterified fatty acid levels were measured as previously described (Takada *et al*. 2013, 2014; Ono *et al*. 2015).

### Plasma concentrations of sesamin and SC‐1

Plasma samples were extracted after hydrolysis with β‐glucuronidase/arylsulfatase. Sesamin and SC‐1 were measured by ultraperformance liquid chromatography–tandem mass spectrometry (UPLC‐MS/MS) as previously described (Tomimori *et al*. 2013).

### Intraperitoneal glucose and insulin tolerance tests

For the glucose or insulin tolerance test, mice were fasted for 6 h and were given an i.p. injection of glucose (1 mg g^−1^) or human insulin (0.25 mU g^−1^) in purified water. Blood samples were repeatedly drawn from the tail vein of the same mice before and 15, 30, 60, 90 and 120 min after the injection. Blood glucose levels were determined using a glucometer (Glutest Ace R; Sanwa Kagaku Kenkyusho, Nagoya, Japan).

### Treadmill testing with expired gas analysis and spontaneous physical activity

Mice were treadmill tested to measure indexes defining exercise capacity as previously described (Kinugawa *et al*. 2005; Yokota *et al*. 2009; Takada *et al*. 2013, 2014; Suga *et al*. 2014; Kadoguchi *et al*. 2015; Nishikawa *et al*. 2015). At the time of treadmill testing, each mouse was placed on a treadmill enclosed by a metabolic chamber, through which air flowing at a constant speed (1 l min^−1^) was passed (Oxymax 2; Columbus Instruments, Columbus, OH, USA). Oxygen and carbon dioxide gas fractions were monitored at both the inlet and output ports of the metabolic chamber. Basal measurements were obtained over a period of 10 min. Mice were then provided with a 10 min warm‐up period at 6 m min^−1^ with the ramp at 0 deg slope. After animals had warmed up, the angle was fixed at 10 deg, and the speed was increased incrementally by 2 m min^−1^ every 2 min until the mouse reached exhaustion. Exhaustion was defined as spending time (10 s) on the shocker plate (shock grid stimulus area, 51 mm × 51 mm; stimulus current range, 0.34–1.5 mA; stimulus voltage, 163 V) without attempting to re‐engage the treadmill. Whole‐body oxygen uptake and carbon dioxide production were automatically calculated every 10 s by taking the difference between the inlet and output gas flow. The respiratory exchange ratio (RER) was calculated as carbon dioxide production divided by oxygen uptake. Work was defined as the product of the vertical running distance and body weight. Spontaneous physical activity was measured using an animal movement analysis system (ACTIMO System; Shintechno, Fukuoka, Japan) as previously described (Takada *et al*. 2013).

### Mitochondrial enzyme activities in the skeletal muscle

The enzymatic activity of citrate synthase (CS), a key enzyme of tricarboxylic acid cycle, was determined spectrophotometrically in the tissue homogenates from skeletal muscle samples, as described previously (Inoue *et al*. 2012; Suga *et al*. 2014; Takada *et al*. 2014; Kadoguchi *et al*. 2015; Nishikawa *et al*. 2015).

### Immunoblotting in the skeletal muscle

Immunoblotting was performed using antibodies against the phosphorylated forms of AMPK and acetyl‐CoA carboxylase‐β (Cell Signaling, Danvers, MA, USA). Equal loading of protein was verified by immunoblotting with glyceraldehyde 3‐phosphate dehydrogenase (GAPDH; Cell Signaling), as previously described (Takada *et al*. 2013; Fukushima *et al*. 2014; Kadoguchi *et al*. 2015; Nishikawa *et al*. 2015; Ono *et al*. 2015).

### Quantitative RT‐PCR

Total RNA was extracted from the hindlimb skeletal muscle limb in the four groups of mice using QuickGene‐810 (Fujifilm, Tokyo, Japan) according to the manufacturer's instructions. Complementary DNA was synthesized with a high‐capacity cDNA reverse transcription kit (Applied Biosystems, Foster City, CA, USA) as previously described (Takada *et al*. 2013). TaqMan quantitative PCR was performed with the 7300 real‐time PCR system (Applied Biosystems) to amplify samples for fatty acid binding protein 3 (*Fabp3*), fatty acid transport protein 1 (*Fatp1*), FAT/CD36 (*Cd36*), carnitine palmitoyltransferase‐1b (*Cpt‐1b*), superoxide dismutase (*Sod1*), *Sod2*, *Catalase*, glutathione peroxidase (*Gpx*), sirtuin 1 (*Sirt1*), peroxisome proliferator‐activated receptor γ coactivator 1α (*Pgc‐1*), nuclear respiratory factor‐1 (*Nrf‐1*) and mitochondrial transcription factor A (*Tfam*). Relative mRNA was analysed with the ΔΔ threshold cycle (ΔΔ*C*_T_) method and normalized to GAPDH as the internal control. Each *C*_T_ value was determined by subtracting the GAPDH RNA *C*_T_ value from the target gene *C*_T_ value. The *C*_T_ value was calculated by subtracting the *C*_T_ value of ND mice from the *C*_T_ value of ND+Sesamin, HFD and HFD+Sesamin mice. The value $2^{-\Delta \Delta C_{T}}$ represented the average relative amount of mRNA for each target gene as previously described (Takada *et al*. 2013).

### Superoxide anion and NAD(P)H oxidase activity in the skeletal muscle *in vivo*

The chemiluminescence elicited by O_2_·^−^ in the presence of lucigenin (5 μmol l^−1^) was measured in hindlimb skeletal muscle using a luminometer (AccuFLEX Lumi 400; Aloka, Tokyo, Japan) as previously described (Yokota *et al*. 2009; Takada *et al*. 2013, 2014; Fukushima *et al*. 2014; Suga *et al*. 2014; Kadoguchi *et al*. 2015; Nishikawa *et al*. 2015; Ono *et al*. 2015). NAD(P)H oxidase activity was measured in the homogenates isolated from hindlimb skeletal muscle by the lucigenin assay after the addition of NAD(P)H (300 μmol l^−1^) as previously described (Yokota *et al*. 2009; Takada *et al*. 2013, 2014; Fukushima *et al*. 2014; Suga *et al*. 2014; Kadoguchi *et al*. 2015; Nishikawa *et al*. 2015; Ono *et al*. 2015).

### NAD(P)H oxidase activity in C2C12 myotubes *in vitro*

The mouse C2C12 myoblast cell line (American Type Culture Collection, Manassas, VA, USA) was seeded on culture plates with medium containing 2% horse serum. Differentiation of C2C12 myoblasts into myotubes occurred in 6–7 days, as confirmed by light microscopy showing morphological alignment, elongation and fusion, as previously described (Fukushima *et al*. 2014; Nishikawa *et al*. 2015). After pre‐incubation at 37°C in serum‐free conditions, C2C12 myotubes were incubated at 37°C with with 1 μmol l^−1^ angiotensin II (Sigma‐Aldrich) for 24 h in the absence or presence of 1 or 10 μmol l^−1^ SC‐1, which was prepared as previously described (Urata *et al*. 2008). After 24 h of incubation, cells were harvested and stored at −80°C for measurement of NAD(P)H oxidase activity. The NAD(P)H oxidase activity was measured in the homogenates of the C2C12 myotubes by a lucigenin (5 μmol l^−1^) assay after the addition of NAD(P)H (100 μmol l^−1^) as previously described (Fukushima *et al*. 2014; Nishikawa *et al*. 2015).

### Statistical analysis

Data are expressed as means ± SEM. For multiple‐group comparisons, two‐way ANOVA followed by Tukey's test was performed. In i.p. glucose and insulin tolerance tests, differences between groups were determined with repeated‐measures ANOVA. A value of *P* < 0.05 was considered statistically significant.

## Results

### Animal characteristics

**Figure 1:**
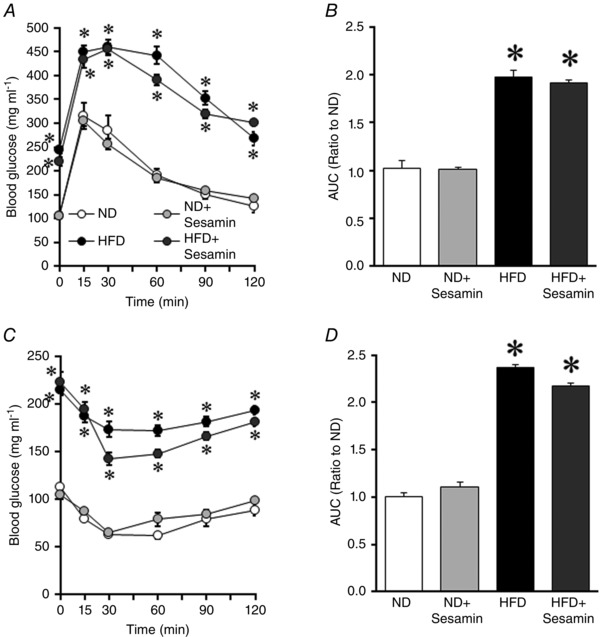
**Glucose tolerance test and insulin tolerance test** Blood glucose levels during i.p. glucose tolerance test (*A*) and insulin tolerance test (*C*) in the normal diet (ND), ND+Sesamin, high‐fat diet (HFD) and HFD+Sesamin mice (*n* = 9–10 for each group). Area under the curve (AUC) of blood glucose levels during i.p. glucose tolerance test (*B*) and insulin tolerance test (*D*) in the ND, ND+Sesamin, HFD and HFD+Sesamin mice. Data are shown as means ± SEM. Experiments were performed after 8 weeks of feeding in all groups. *P < 0.01 *versus* ND.

Table 1 shows the characteristics of the animals in the four groups. Body weight was significantly higher in HFD compared with ND mice, and this was accompanied by a significant increase in the epididymal fat weight (Table 1). There were no differences in the quadriceps, gastrocnemius and soleus muscle weights, systolic blood pressure or heart rate between ND and HFD mice (Table 1). Fasting blood glucose, insulin, triglyceride, total cholesterol and non‐esterified fatty acid levels were significantly higher in HFD mice (Table 2). Moreover, blood glucose levels during the i.p. glucose and insulin tolerance tests were significantly higher in HFD than in ND mice (Fig. 1).

**Table 1:** Animal characteristics

		ND+Sesamin		HFD+Sesamin
Parameter	ND group (*n* = 10)	group (*n* = 10)	HFD group (*n* = 10)	group (*n* = 10)
Haemodynamic measurements				
Systolic blood pressure (mmHg)	101 ± 1	101 ± 2	99 ± 1	101 ± 2
Heart rate (beats min^−1^)	621 ± 40	610 ± 24	613 ± 42	603 ± 19
Body and organ weights				
Body weight (g)	30.3 ± 0.5	29.5 ± 0.2	43.1 ± 0.7*	37.5 ± 1.0*, †
Fat weight (mg)	630 ± 64	460 ± 37	2241 ± 68*	2006 ± 109*
Quadriceps muscle weight (mg)	221 ± 5	234 ± 4	225 ± 3	222 ± 4
Gastrocnemius muscle weight (mg)	171 ± 3	172 ± 3	184 ± 2	170 ± 2
Soleus weight (mg)	11.0 ± 0.2	11.8 ± 0.2	14.4 ± 0.2	12.7 ± 0.3

Data are expressed as means ± SEM. *P < 0.05 *versus* ND; ^†^ *P*<0.05 *versus* HFD. Abbreviations: HFD, high‐fat diet; and ND, normal diet.

**Table 2:** Blood measurements

Parameter	ND group (*n* = 8)	ND+Sesamin group (*n* = 8)	HFD group (*n* = 8)	HFD+Sesamin group (*n* = 8)
Fasting glucose (mg dl^−1^)	106 ± 6	106 ± 5	244 ± 7*	220 ± 10*
Insulin (ng ml^−1^)	0.47 ± 0.09	0.81 ± 0.10	1.96 ± 0.32*	0.76 ± 0.16†
Triglyceride (mg ml^−1^)	40 ± 2	45 ± 7	82 ± 4*	54 ± 6†
Total cholesterol (mg ml^−1^)	74 ± 6	55 ± 4	198 ± 6*	81 ± 15†
Non‐esterified fatty acid (mequiv l^−1^)	0.25 ± 0.02	0.20 ± 0.03	0.87 ± 0.02*	0.37 ± 0.05†

Data are expressed as means ± SEM. *P < 0.05 *versus* ND; ^†^ *P *< 0.05 *versus* HFD.

Plasma sesamin and SC‐1 were detected in ND+Sesamin and HFD+Sesamin mice (sesamin, 380 ± 354 and 323 ± 186 nmol l^−1^; and SC‐1, 1.00 ± 0.43 and 1.85 ± 0.43 μmol l^−1^). Sesamin significantly suppressed an increase of body weight, but did not affect haemodynamic measurements (Table 1). There were also no significant differences in epididymal fat weight, total skeletal muscle weight, fasting blood glucose or blood glucose levels between HFD and HFD+Sesamin mice during the i.p. glucose tolerance test (Tables 1 and 2 and Fig. 1). In contrast, the increases in plasma insulin, triglyceride, total cholesterol and non‐esterified fatty acid levels were completely attenuated in HFD+Sesamin mice. These results showed that HFD feeding for 8 weeks induced type 2 diabetes with the characteristics of obesity and glucose intolerance, and that sesamin prevented the increases in blood insulin and serum lipid levels.

### Exercise capacity and spontaneous physical activity

**Figure 2:**
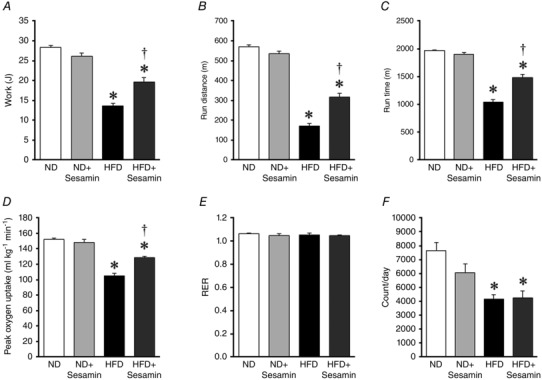
**Exercise capacity and spontaneous physical activity** The summarized data of the work (*A*), distance run (*B*), run time (*C*), peak oxygen uptake (*D*), respiratory exhange ratio (RER) to exhaustion (*E*) and spontaneous physical activity (*F*) in the ND, ND+Sesamin, HFD and HFD+Sesamin mice (*n* = 10 for each group). Data are shown as means + SEM. *P < 0.05 *versus* ND; ^†^ *P* < 0.05 *versus* HFD.

Figure 2 shows the indices of exercise capacity. The work, run distance and run time to exhaustion were significantly decreased in HFD compared with ND mice. The reduced exercise capacity was partly ameliorated in HFD+sesamin mice (Fig. 2 *A–D*). In contrast, spontaneous physical activity was significantly decreased in HFD compared with ND mice, and this effect was not altered by sesamin (Fig. 2 *F*).

### Mitochondrial function and biogenesis in the skeletal muscle

**Figure 3:**
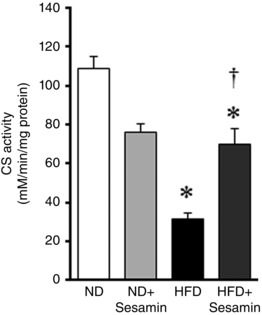
**CS activity** The summarized data for citrate synthase (CS) in the skeletal muscle from the ND, ND+Sesamin, HFD and HFD+Sesamin mice (*n* = 6 for each group). Data are shown as means + SEM. *P < 0.05 *versus* ND; ^†^ *P* < 0.05 *versus* HFD.

**Figure 4:**
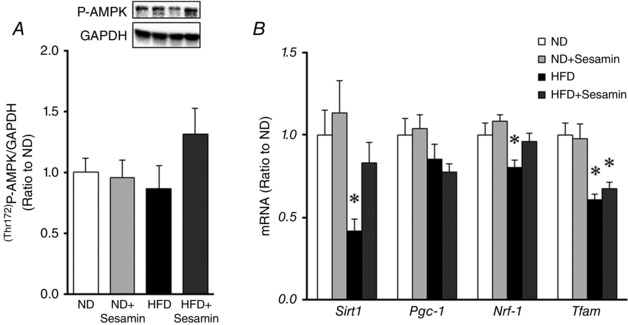
**Mitochondrial biogenesis related‐protein and gene expressions** Quantitative analysis of the phosphorylated form (Thr172) of AMP kinase (AMPK) protein (*A*) and gene expressions of sirtuin1 (*Sirt1*), peroxisome proliferator‐activated receptor γ coactivator 1 (*Pgc‐1*), nuclear respiratory factor 1 (*Nrf‐1*) and mitochondrial transcription factor A (*Tfam*) mRNA (*B*) in skeletal muscle obtained from the ND, ND+Sesamin, HFD and HFD+Sesamin mice (*n* = 6–8 for each group). Protein expression was normalized to glyceraldehyde 3‐phosphate dehydrogenase (GAPDH). Gene expressions were normalized to GAPDH and depicted as the ratio to ND. Data are shown as means + SEM. *P < 0.05 *versus* ND.

Exercise capacity is largely dependent on mitochondrial function in the skeletal muscle. It has been reported that the activity of CS, a key enzyme of the tricarboxylic acid cycle, in the skeletal muscle plays a critical role in exercise capacity (MacDougall *et al*. 1998; Kanatous *et al*. 1999; Park *et al*. 2014). Moreover, CS activity closely is related to mitochondrial quantity, complex activities and respiration in the permeabilized muscle fibre (Kanatous *et al*. 1999; Park *et al*. 2014). Therefore, the activity of this mitochondrial enzyme was measured (Fig. 3). Skeletal muscle CS activity was significantly decreased in HFD compared with ND mice, and this decrease was reversed by sesamin. In contrast, there were no differences between groups in the phosphorylation of the mitochondrial biogenesis‐related protein AMPK or in the gene expression of *Pgc‐1* mRNA (Fig. 4). Gene expressions of *Sirt1*, *Nrf‐1* and *Tfam* mRNA were significantly decreased in HFD compared with ND mice, and these differences were not affected by sesamin (Fig. 4).

### Fatty acid oxidation and glucose metabolism in the skeletal muscle

**Figure 5:**
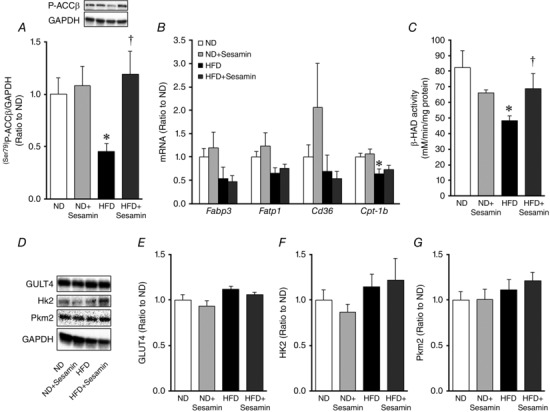
**Substrate metabolism related‐protein and gene expressions** Summarized data of quantitative analysis of the phosphorylated form (Ser79) of acetyl‐CoA carboxylase‐β (ACCβ) protein (*A*), gene expressions of fatty acid binding protein 3 (*Fabp3*), fatty acid transport protein 1 (*Fatp1*), *Cd36* (FAT/CD36) and carnitine palmitoyltransferase‐1b (*Cpt‐1b*) mRNA (*B*) and β‐hydroxyacyl‐CoA dehydrogenase (β‐HAD) activity (*C*) in skeletal muscle obtained from the ND, ND+Sesamin, HFD and HFD+Sesamin mice (*n* = 6–8 for each group). Representative bands (*D*) and quantitative analysis of protein expressions of glucose transporter 4 (GLUT4; *E*), hexokinase 2 (Hk2; *F*) and pyruvate kinase m2 (Pkm2; *G*) in the skeletal muscle obtained from ND, ND+Sesamin, HFD and HFD+Sesamin mice (*n* = 6 for each group). Data are shown as means + SEM. *P < 0.05 *versus* ND; ^†^ *P* < 0.05 *versus* HFD.

Acetyl‐CoA carboxylase‐β phosphorylation and β‐hydroxyacyl‐CoA dehydrogenase activity in the skeletal muscle were significantly decreased in HFD compared with ND mice, and these decreases were inhibited by sesamin (Fig. 5 *A* and *C*). *Cpt‐1b* mRNA was significantly decreased in the skeletal muscle from HFD compared with ND mice, but this difference was not affected by sesamin (Fig. 5 *B*). *Fabp3*, *Fatp1* and *Cd36* mRNA also tended to be decreased in HFD mice and were not affected by sesamin. In contrast, there were no differences in glucose transporter 4, hexokinase 2 and pyruvate kinase m2 among the four groups (Fig. 5 *D–G*).

### Oxidative stress in the skeletal muscle

**Figure 6:**
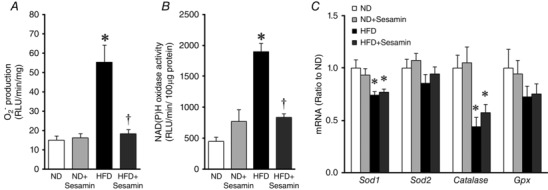
**Oxidative stress and antioxidant capacity** Superoxide (O_2_·^−^; *A*) and NAD(P)H oxidase activity (*B*) measured by lucigenin chemiluminescence in the skeletal muscle obtained from ND, ND+Sesamin, HFD and HFD+Sesamin mice (*n* = 6–10 for each group). Quantitative gene expressions of superoxide dismutase 1 (*Sod1*), *Sod2*, *Catalase* and glutathione peroxidase (*Gpx*) mRNA (*C*) in skeletal muscle obtained from the ND, ND+Sesamin, HFD and HFD+Sesamin mice (*n* = 8 for each group). Data are shown as means + SEM. Abbreviation: RLU, relative light units. *P < 0.05 *versus* ND; ^†^ *P* < 0.05 *versus* HFD.

**Figure 7:**
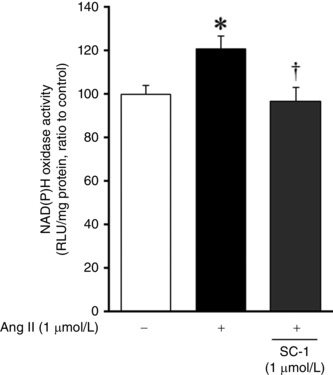
**Effect of SC‐1 on NAD(P)H oxidase activity** NAD(P)H oxidase activity measured by lucigenin chemiluminescence in the C2C12 myotubes obtained from the control, angiotensin II (Ang II) 1 μm and Ang II+SC‐1 (1 μm) groups (*n* = 10–11 for each group). Data are shown as means + SEM. Abbreviation: RLU, relative light units. *P < 0.05 *versus* ND; ^†^ *P* < 0.05 *versus* HFD.

Superoxide anion production and NAD(P)H oxidase activity were significantly increased in the skeletal muscle from HFD compared with ND mice, and this change was completely inhibited by sesamin (Fig. 6 *A* and *B*). Moreover, SC‐1 at a dose of 1 μmol l^−1^ significantly suppressed an increase in NAD(P)H oxidase activity by angiotensin II stimulation in C2C12 myotubes (Fig. 7). *Sod1* and *Catalase* mRNA in the skeletal muscle were significantly decreased in HFD compared with ND mice, but were not affected by sesamin (Fig. 6 *C*).

## Discussion

In the present study, mice with HFD‐induced diabetes exhibited lowered exercise capacity, decreased activity of the enzyme CS and activation of NAD(P)H oxidase in the skeletal muscle; all these effects were significantly ameliorated by chronic oral administration of sesamin to HFD mice. Therefore, dietary sesamin was shown to prevent the decline in exercise capacity and the impairment of mitochondrial function in mice with HFD‐induced diabetes.

### Effects of sesamin on substrate metabolism

Sesamin has hypoglycaemic and hypolipidaemic effects in diabetic mice (Nakano *et al*. 2006, 2008; Hong *et al*. 2013). Hong *et al*. (2013) reported that sesamin decreased blood glucose, insulin and lipid levels in mice with type 2 diabetes. Sesamin also increased hepatic CPT activity in mice or fat oxidation in rats (Shimoda *et al*. 2006; Ide *et al*. 2009). Coinciding with these results, in the present study sesamin prevented the impairment in lipid metabolism in the skeletal muscle (Fig. 5) and, consequently, decreased blood lipids in HFD mice (Table 2). In contrast, it did not affect the fasting glucose level, glucose and insulin tolerance (Table 2 and Fig. 1) or glucose metabolism in the skeletal muscle (Fig. 4). Therefore, these results suggest that sesamin specifically maintains the lipid substrate metabolism and may have beneficial effects on energy production in the skeletal muscle.

### Effects of sesamin on exercise capacity and NAD(P)H oxidase activity

The most significant finding of the present study was that chronic administration of sesamin to HFD mice prevented the decrease in exercise capacity (Fig. 2) and the impairment of mitochondrial function, including activity of the tricarboxylic acid cycle (Fig. 3). In contrast, sesamin did not affect the signalling associated with mitochondrial biogenesis (Fig. 4). These results show that sesamin maintains mitochondrial function without changing the number of mitochondria in the skeletal muscle.

Our previous papers showed that NAD(P)H oxidase‐induced O_2_·^−^ production impaired mitochondrial function in the skeletal muscle of HFD mice (Yokota *et al*. 2009; Takada *et al*. 2013; Suga *et al*. 2014). However, the mechanisms for mitochondrial dysfunction induced by NAD(P)H oxidase‐dependent O_2_·^−^ in skeletal muscle are not fully understood. The decrease of mitochondrial electron transport chain complex I and III activities can potentially be explained by direct oxidative damage to mitochondrial complexes (Doughan *et al*. 2008; Yokota *et al*. 2009). Mitochondria can be the primary target for oxidative damage when production of reactive oxygen species exceeds the capacity of the endogenous reactive oxygen species scavenging system. Superoxide anion easily impairs these electron transport chain complexes because they include an iron–sulfur centre. Multiple iron–sulfur centres exist in complexes in complex I and II. In mice lacking superoxide dismutase, destruction of the iron–sulfur centres in the mitochondria has been described (Li *et al*. 1995; Morten *et al*. 2006). In addition, oxidative damage to mitochondrial DNA can also result in a decrease of electron transport chain complex activities (Ide *et al*. 2001 *b*). Furthermore, impaired mitochondrial DNA may adversely affect mitochondrial biogenesis. In contrast, given that sesamin has an antioxidant effect, we hypothesized that it may have a favourable effect on mitochondrial function in HFD‐induced diabetic mice.

Several studies have reported that sesamin increases superoxide dismutase, catalase and glutathione peroxidase activities in the liver or aortic tissue (Roghani *et al*. 2011; Hong *et al*. 2013). In the present study, skeletal muscle expressions of antioxidant genes, in particular *Sod1* and *Catalase* mRNA, were significantly decreased in HFD mice, and sesamin did not affect them (Fig. 6 *C*). Therefore, sesamin attenuated oxidative stress without affecting the antioxidant enzymes in the skeletal muscle of HFD mice.

### Effects of SC‐1 on inhibition of NAD(P)H oxidase activity

Direct inhibition of the activation of NAD(P)H oxidase by sesamin or SC‐1 may be associated with the present results as another possible mechanism. It has been reported that oral sesamin (1% w/w) feeding attenuated deoxycorticosterone acetate‐ and salt‐induced increases in NAD(P)H‐dependent O_2_·^−^ production in the rat aorta (Nakano *et al*. 2008). In a previous study investigating the metabolic pathway of sesamin, it has been shown that the methylenedioxyphenyl moiety in the structure of sesamin is changed into a dihydroxyphenyl (catechol) moiety in the liver (Nakai *et al*. 2003). SC‐1, one of the metabolites of sesamin, was shown to inhibit O_2_·^−^ production in rat aorta (Nakano *et al*. 2006). In the present study, after 8 weeks of feeding sesamin, plasma concentrations of SC‐1 were 1.00 ± 0.43 and 1.85 ± 0.43 μmol l^−1^ in the ND+Sesamin and HFD+Sesamin mice, respectively. SC‐1 at a dose of 1.0 μmol l^−1^ significantly inhibited NAD(P)H oxidase activity induced by angiotensin II stimulation in C2C12 myotubes (Fig. 7). Therefore, SC‐1 may have a direct inhibitory effect on NAD(P)H oxidase activity in HFD‐induced diabetic mice.

### Clinical implications

The incidence of type 2 diabetes has been increasing markedly, creating both medical and social challenges in industrialized countries. Our present data showed that supplemental treatment with sesamin prevented the increases in insulin and lipid levels and the decline in exercise capacity in type 2 diabetic mice. Given the close association between exercise capacity and prognosis, sesamin could be useful for treatment of type 2 diabetes in humans.

### Conclusion

Sesamin prevented the decline in exercise capacity in mice with HFD‐induced diabetes by maintenance of mitochondrial function, fat oxidation and attenuation of oxidative stress in the skeletal muscle. Our data suggest that sesamin would contribute novel ameliorating activities to the treatment of diabetes mellitus, especially by improving patients’ lowered exercise capacity.

## Additional information

### Competing interests

This study was funded by Suntory Wellness Ltd, and D.T., Y.O. and H.S. are employees of Suntory Wellness Ltd. The sponsor had no control over the interpretation, writing or publication of this work. The corresponding author had full access to all the data in the study and had final responsibility for the decision to submit for publication.

### Author contributions

S.T. designed experiments, performed experiments, analysed data and wrote the manuscript. S.K. conceived and designed experiments and wrote the manuscript. T.F., W.M. and A.F. performed experiments, analysed data and wrote the manuscript. D.T., Y.O. and H.S. performed experiments, analysed data and contributed to discussions. S.M., T.Y., K.O. and H.T. designed experiments, contributed to discussions and reviewed and edited the manuscript. All authors have read and approved the manuscript.

### Funding

This study was supported by grants from the Ministry of Education, Culture, Sports, Science and Technology (24390192, 25893005, 26350879, 26750331, 15K01625, 15K09115, 15K19358 and 15H04815).
